# On the Coexistence of Captions and Sign Language as Accessibility Solutions in Educational Settings

**DOI:** 10.3390/audiolres16010020

**Published:** 2026-01-29

**Authors:** Francesco Pavani, Valerio Leonetti

**Affiliations:** 1 Center for Mind/Brain Sciences (CIMeC), University of Trento, 38068 Rovereto, Italy; 2 Centro Interuniversitario di Ricerca “Cognizione, Linguaggio e Sordità” (CIRCLeS), University of Trento, 38068 Rovereto, Italy; 3 Azienda Ospedaliero, Universitaria di Cagliari, 09124 Cagliari, Italy; leonettivalerio@yahoo.it; 4 Department of Medical Science and Public Health, University of Cagliari, 09124 Cagliari, Italy

**Keywords:** deaf, hard-of-hearing, captions, sign language, accessibility

## Abstract

**Background/Objectives:** In mainstream educational settings, deaf and hard-of-hearing (DHH) students may have limited or no access to the spoken lectures and discussions that are central to the hearing majority classroom. Yet, engagement in these educational and social exchanges is fundamental to their learning and inclusion. Two primary visual accessibility solutions can support this need: real-time speech-to-text transcriptions (i.e., captioning) and high-quality sign language interpreting. Their combined use (or coexistence), however, raises concerns of competition between concurrent streams of visual information. This article examines the empirical evidence concerning the effectiveness of using both captioning and sign language simultaneously in educational settings. Specifically, it investigates whether this combined approach leads to better or worse content learning for DHH students, when compared to using either visual accessibility solution in isolation. **Methods:** A review of all English language studies in peer-reviewed journals until August 2025 was performed. Eligible studies used an experimental design to compare content learning when using sign language and captions together, versus using sign language or captions on their own. **Databases Reviewed:** EMBASE, PubMed/MEDLINE, and PsycInfo. **Results:** A total of four studies met the criteria for inclusion. This limited evidence is insufficient to decide on the coexistence of captioning and sign language. Yet, it underscores the potential of captions for content access in education for DHH, even when sign language is available. **Conclusions:** The present article reveals the lack of evidence in favor or against its coexistence with sign language. With the aim to be constructive for future research, the discussion offers considerations on the attentional demands of simultaneous visual accessibility resources, the diversity of DHH learners, and the impact of current and forthcoming technological advancements.

## 1. Introduction

For students who are deaf or hard-of-hearing (DHH) and attend mainstream education systems, accessing spoken content and classroom conversations can be a significant challenge. This applies not only to lecture information provided by the teacher, but also to discussions and questions raised by hearing classmates: engaging in these academic and social exchanges is fundamental for learning and inclusion. Methodologies and pedagogical approaches that promote achievements for all learners can also favor students who are DHH. For instance, pacing of instructions, teacher expectations, and classroom management are among the factors that can positively impact learning outcomes [1]. Likewise, the teacher’s ability to deliberately incorporating pauses into the lecture (called “wait time” [2] can promote a better discourse structure for learning, encouraging more student-driven speech [3]. Furthermore, the individual and collective sense of the efficacy of teachers (i.e., the belief that teachers have in their capacity as a single person or as a group to successfully plan and take actions that can have positive effects on students) can favor hearing as well as DHH students [4].

However, to address language and content accessibility challenges, learning environments for DHH students should also offer specific solutions, such as real-time speech-to-text transcriptions (captioning), visual aids (slides), written lecture summaries (notes), or sign language interpreting. These accessibility solutions could, in principle, complement each other—i.e., they could coexist within the same educational setting. However, practical considerations such as costs or organizational constraints related to each of the accessibility solutions could lead institutions to prioritize one over the other, or to decide on some specific combinations (e.g., captioning and slides) but not others. This contribution examines the evidence supporting the simultaneous combination of two accessibility solutions: captioning and sign language interpreting.

Broadly speaking, the coexistence of these two accessibility solutions in educational settings typically raises two types of concerns. The first relates to the acceptance and appropriateness of sign language in these learning environments. As Knoors and Marschark clearly state ([5], p. 7), while there is consensus on the fact that “language is an essential ingredient for normal development, learning, and academic achievement”, the issue of the relative contribution of sign language and spoken language is “rarely discussed with the *or* explicitly included, even if it frequently is implicit”. In other words, the long-standing debate in deaf education [6] still polarizes discussions between positions advocating for sign language for all DHH learners [7,8] and persistent skepticism, albeit scientifically ungrounded [9,10,11]. The shift away from sign language is especially evident in several scenarios: when deaf students are enrolled in mainstream educational settings unfamiliar with sign language; when the DHH student’s hearing family has no previous knowledge of sign language; or when considering DHH students who use assisted-hearing devices (hearing aids or cochlear implants). In the perspective of many parents, educators, or influential experts (including clinicians), these technological advancements have rendered sign language unnecessary for several DHH students (but see [12] for careful considerations of the impact of cochlear implantation in DHH education, and the importance of providing a range of choices to meet the needs of an increasingly diversified population of DHH students).

**Figure 1:**
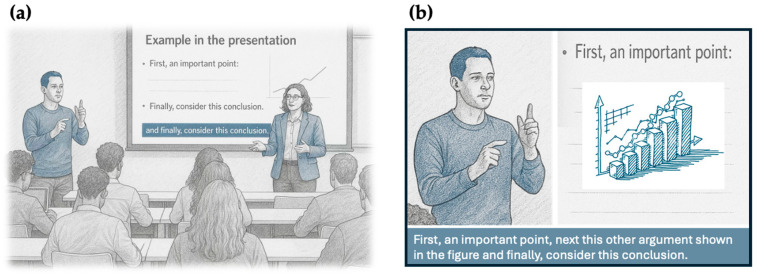
Two hypothetical scenarios of coexistence of captions with sign language interpreting in the educational context. (**a**) A classroom where multiple sources of content information are distributed across different spatial regions (the speaking teacher, the captions at the bottom of the projected slide, the sign language interpreter, all highlighted in light blue). In this setting, students who wish to attend to each of the visual information need to move their attention, but also their gaze, across different areas of the lecture room. (**b**) A more integrated scenario where the sign language interpreter, captions, and slides contents are positioned closer together. This minimizes the need for large gaze shifts, and yet it does not eliminate the attentional competition between the different sources of information.

The second concern relates to the risk of cognitive load involved in accessing multiple streams of information at once. Imagine a mainstream lecture like the one depicted in Figure 1 below. In panel A, a student attends a lecture in a room where the teacher, the projected slides, the captions, and the sign language interpreter are all in different portion of the room. Or consider the relatively better scenario depicted in panel B, where all these pieces of information are spatially integrated within the area of a screen. In either case, extracting information from the different sources of information implies continuous shifts in visual attention, possibly with head and/or eye movements when the sources of information are more disparate (Figure 1a). As originally proposed in the *split-attention hypothesis* within the cognitive theory of multimedia learning [13], when multiple visual materials are concurrently presented (e.g., printed words and animations, in the original studies conducted with typical hearing participants), this could prevent adequate attention to some of the presented materials (see also [14]). This may result in a more fragmented perceptual and attention experience, increasing the likelihood of missing key information. In addition, it contributes to greater cognitive load and mental effort for the student. The essence of this concern is that adding multiple sources of visual information might hinder rather than support learning.

In the present article, we examine whether the existing empirical evidence indicates that the coexistence of these accessibility solutions leads to better (or worse) content learning for deaf and hard-of-hearing (DHH) students in educational settings, compared to using each solution alone. To this aim, we conducted a review of all English language studies in peer-reviewed journals until August 2025. To anticipate, the results showed that the current evidence is too scarce and inconclusive to be used for practical implementation in educational settings. Yet, some broad suggestions appear to emerge and, most importantly, research priorities for advancing decisions on accessibility in this domain can be envisioned. In the discussion, we highlight these suggestions and detail the most evident research priorities focusing on the attentional demands of simultaneous visual accessibility resources, the diversity of DHH learners, and the impact of technological advancements. While these considerations should be considered speculative considering the current state of the evidence, we hope that they could help structure future directions for research.

## 2. Methods

### 2.1. Search Criteria

A review of the literature was performed. The review aimed to explore content learning scores (outcome), following exposure to educational materials delivered with sign language and captions combined versus sign language or captions alone (comparison), in deaf or hard-of-hearing students (population). To identify studies for inclusion in this review, detailed search strategies were developed for the following three databases: PubMed (U.S. National Library of Medicine, National Institutes of Health, Bethesda, MD, USA), EMBASE (Elsevier, Amsterdam, Netherlands), and PsycInfo (American Psychological Association, Washington, DC, USA). Search strategies were developed using combinations of title/abstract keywords and MeSH terms. Detailed search strings for each database are reported in Table 1.

### 2.2. Data Extraction

Each database was search up to August 2025. Data extraction was performed by V.L. on three databases. All retrieved records were then exported in the review management software Rayyan [15] for removing duplicates, labeling of exclusion criteria, study selection, and full-text analysis.

### 2.3. Selection Criteria

Studies were considered for inclusion if they (1) were empirical studies; (2) were conducted on deaf or hard-of-hearing students; (3) measured content learning in educational settings; (4) compared educational materials delivered with sign language and captions combined versus sign language or captions alone. Exclusion criteria included the following: duplicates, non-English language, non-human studies, review articles, commentaries, case reports, inaccessible full-text articles, incomplete or missing statistical data. To counteract the risk of bias, the Rayyan software was utilized as a platform to support the assessment process. Its features allowed for blinded, independent review, structured conflict resolution, and the upload and annotation of full-text articles as evidence for the judgments. Abstracts were first independently and blindly assessed by the two authors to identify all articles that met the inclusion criteria. Conflicts were resolved by discussions until consensus was reached.

## 3. Results

**Figure 2:**
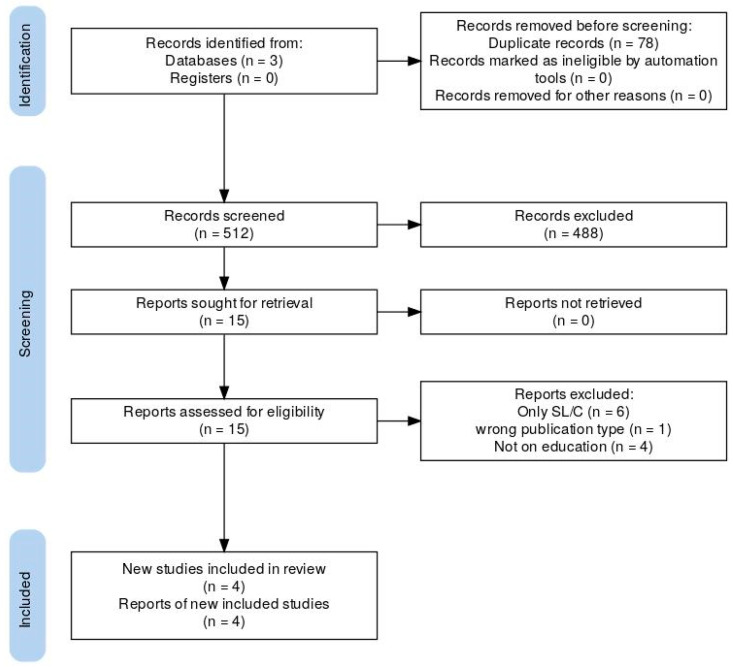
Article identification, screening, and inclusion flow-chart.

A total of 512 records were collected in the search after the removal of duplicates, which were all screened by title and abstract. This resulted in 488 exclusions, nine conflicts, and 15 inclusions. Full-text reviews of the included articles resulted in four articles being selected for the final examination, all published between 2004 and 2022. This search process is summarized in Figure 2 using the PRISMA [16] flow-chart for clarity, albeit our figure does not configure it as a systematic review.

### Qualitative Description of the Studies Selected for Final Inclusion

The four selected studies are reported in Table 2 and Table 3, in chronological order. For each study, we report (when available) the participant’s numerosity, the degree of hearing loss, the use of sign language by the participants, assessment of reading proficiency, mean age and range, context, type of stimuli, experimental conditions used in the design and the statistical comparisons (independent variables), the measure (dependent variable), the outcome, and an estimate of the effect size based on the reported statistics.

Gentry and colleagues [17] compared the ability to retell contents (stories) delivered through videos under four within-participants conditions: with print only, with print + pictures, with print + sign language, or with all visual stimuli combined (print + picture + sign language). Participants were DHH adolescents (N = 25, mean age 12.3 years old), all from mainstream schools (except three from residential schools for deaf students). All are described in the article as “deaf students” but the actual degree of hearing loss is not reported. Yet, they all used sign language as their primary communication mode. They were included if their reading level corresponded to three-to-fourth grade, as determined by the Stanford Achievement Test for the Hearing-Impaired (SAT-HI). Participants retold the story using sign language. The conditions in which the story was narrated using pictures (i.e., print + pictures, or print + pictures + sign language) led to better performance compared to the conditions in which pictures were absent (i.e., print only, or print + sign language), with a medium to large effect size explaining ~48% of the variance.

Marschark and colleagues [18] addressed the issue in a series of four experiments. They asked participants to attend introductory-level video-recorded lectures and tested their learning through multiple-choice questions afterwards. In experiments 1 and 2, they involved DHH university students (Exp. 1, N = 95; Exp. 2, N = 60) and compared their performance with a smaller sample of hearing peers (Exp. 1, N = 32; Exp. 2, N = 20). More than 90% of the DHH participants experienced profound deafness (mean hearing threshold was 80 dB in the better ear). All students were recruited from a bilingual institute in which sign language skills are required. Reading proficiency was formally assessed through reading comprehension tests (e.g., the American College Test (ACT) reading comprehension subset).

In experiment 1, they compared between-participants video lectures with a speech-to-text approach based on a condensed representation of speech (known as “C-Print”), with video lectures with sign language interpreting, or video-lectures with the combination of the two methods. They found that learning was better with C-Print compared to interpreting or interpreting plus C-Print, irrespective of the students’ prior experience with C-Print materials (a moderate to large effect size was measured, explaining ~12% of the variance). In experiment 2, they repeated the study introducing also the full verbatim transcription of the spoken content (known as CART, i.e., Communication Access Realtime Translation). They also provided visual displays during the lecture (i.e., power-point presentations) and study notes derived from the speech-to-text services (i.e., C-Print or CART) and tested participants immediately after learning and 1 week after the lecture, allowing in the meantime students to study the notes. In experiment 2, the combined use of speech-to-text and interpreting was no longer examined, and the comparison included only C-Print, CART, or sign language. This time, no obvious difference between speech-to-text services and sign language interpreting emerged.

In experiment 3, they continued to compare learning via condensed real-time text (C-Print) and sign language, but enrolling secondary school students (N = 15, aged 12–16 years old) rather than university students. All 15 students were profoundly deaf (mean hearing threshold was 108 dB in the better ear) and reading proficiency was formally assessed through reading comprehension tests as before. Again, students were recruited from a bilingual institute in which sign language skills are required. Participants were tested on the lecture content both immediately after each lesson and 1 week later, this time without notes. Importantly, unlike experiments 1 and 2, in this additional experiment the classroom lecture was delivered directly in sign language rather than being interpreted from spoken language. The results proved inconclusive, as none of the content delivery methods clearly surpassed the others. It is worth noting that scores across the three delivery modes in experiment 3 were rather low for DHH students (30–45% accuracy range), suggesting that content learning remains challenging for deaf students (as also reported in experiment 1).

Finally, in experiment 4, they recruited a novel group of deaf secondary school students (N = 28, aged 14.9 years on average, all with hearing thresholds above 90 dB in the better ear). This time, contents were delivered through a video that included captioning, or sign language plus captioning. As noted by the authors, here captioning and interpreting were superimposed on the video within a closer area of space (as in typical television programs; as in Figure 1b), whereas in the previous experiment the “students’ gaze would have had to traverse significantly greater distances in order to shift from one source of information to the other” (p. 433). As before, content comprehension was assessed immediately after the video with multiple-choice questions. Although students learned somewhat more novel words and understood more content with the combination of captioning and sign language compared to captioning alone, this effect was small and was not confirmed when the analysis also considered prior content word knowledge of each student. Taken together, the results from Marschark and colleagues [18] outlined neither an advantage (nor a disadvantage) for delivering contents with print materials compared to high-quality sign language. Except for their experiment 1, no specific difficulty related to the combined use of captions and sign language was noted. In addition, while C-Print was found to lead to higher performance in some conditions, it did not consistently outperform other methods like sign language interpreting.

Yoon and Kim [19] studied the effects of adding captions to sign language interpreting for content comprehension of an education video. The effects of this manipulation were also examined on cognitive load and motivation for online learning. Participants in the study were deaf adolescents and adults (N = 62, mean age 23 years old, mean hearing threshold 90 dB or more in the better ear, all reporting sign language as their first language). All participants were screened for reading proficiency using formal assessment (Test of Proficiency in Korean; TOPIK). Half of participants viewed the video’s educational content with a sign language interpreter, whereas the remaining half also saw captions added to the video. Content comprehension was examined with multiple-choice questions, whereas cognitive load and motivation were assessed with Likert scales. A significant benefit for the sign language plus caption condition was observed for content comprehension, with a medium to large effect size explaining ~55% of the variance. Instead, no effect of the manipulation was observed for cognitive load and motivation for online learning. The study identifies several factors—such as the placement of sign language video segments, caption speed, color saturation (chromaticity), and font size—as elements that determine cognitive load. However, it did not explore how these elements interact with each other or their combined effect on material comprehension.

Finally, Almalhy [20] compared three methods to deliver educational content to DHH university students (N = 54, estimated age 17–19 based on education level, mean hearing threshold 71 dB or more in the better ear, none using hearing aid or cochlear implant, all with adequate sign language and reading skills for the task as assessed by the author). Educational contents were delivered through five video tutorials: three focused on the acquisition of declarative knowledge, and two focused on the acquisition of procedural knowledge. Critically, 18 participants saw the videos with captions only, 18 participants with sign language only, and the remaining 18 with captions plus sign language. Comprehension was assessed with multiple-choice questions after each video (immediate) and at the end of each declarative or procedural set of videos (delayed). When declarative knowledge was tested immediately after each video, the captions group performed better than the sign language group, and both performed better than the sign + captions group. Instead, when declarative knowledge was tested after all the videos (delayed) the sign language group and the captions group outperformed the sign + captions group. Notably, the pattern of results changes when procedural knowledge was tested: for both the immediate and delayed tests, the sign language group was better than the caption group, and both outperformed the sign + captions group. Common to the two knowledge learning materials (declarative or procedural) was that the use of sign language or captions alone was better than the simultaneous combination of the two.

## 4. Discussion

### 4.1. Summary of the Results from the Included Studies

We conducted a review to determine if the coexistence of captions and sign language provides a content learning advantage (or disadvantage) for DHH students over the use of a single visual accessibility solution in educational settings. Although the resulting evidence is extremely limited, as is evident from the summary Table 2 and Table 3, some systematicity in the experimental design exists across the selected studies. Specifically, all studies involved DHH participants with severe-to-profound deafness (albeit the exact degree of hearing loss lacked in one study), all studies included participants who were proficient sign language users (as evidenced by their participation in bimodal bilingual education programs, or by self-report), and all studies checked that participants had adequate reading skills for the captions used in the study (primarily using formal testing). In addition, all studies presented the educational content in video format, contrasted the combined use of captions and sign language with one of the two visual accessibility solutions alone, and assessed content learning (primarily through multiple-choice questions).

Despite these similarities across the included studies, our review clearly shows that the empirical evidence addressing the effect of combined captions and sign language for content learning is scarce. This prevents a quantitative meta-analysis of the results and invites great caution to the interpretation and generalizability of the results for educational contexts. With respect to our specific research question, as of now it is not possible to establish whether the combination of sign language plus captions is systematically better or worse compared to either of the visual accessibility solutions alone in educational settings. The pattern of results is too scattered to lead to any unequivocal conclusion. This inconsistency necessitates further investigation into the conditions under which each support service is most effective.

In this context of minimal empirical evidence, any consideration is speculative. The condition that most often resulted in the best content learning appears to be the one that included captions (i.e., [17], experiments 1 and 4 in [18,19], declarative knowledge measure in [20]). If confirmed, this finding would indicate the importance of captions for content access in education for DHH, even when sign language is available and in a context in which sign language was the preferred communication means reported by the student. Also, it would indicate that the assumption that high-quality sign language interpreting alone provides equal access to learning for deaf students should be treated with caution, as it may overlook the importance of captioning. Below, we mention some of the reasons why captions could, theoretically, prove useful in educational contexts. While none of these mechanistic explanations were directly tested in the reviewed studies, we briefly outline them for the benefit of future empirical investigations.

In the three studies that formally assessed reading proficiency, no direct relationship was found between reading skills and access to the educational content via captions. As originally observed by Marschark et al. [18], this is somewhat surprising because one might expect that sign language alone would offer the best possible access for students who are highly fluent signers and considering that (on average) DHH students can present with reading difficulties. The fact that captions still provide benefits may indicate that the key factor may be language fragility rather than reading comprehension itself [18]. In this regard, captions may have introduced a layer of redundancy in the linguistic input, helping DHH students compensate for occasional gaps in sign language understanding.

Another possible explanation for the potential role of captions in the context of sign language interpreting is that educational content is typically delivered in spoken language, with specialized terminology grounded in the written and spoken lexicon of each discipline. Thus, captions can offer direct access to the original linguistic content rather than an interpreted version. Furthermore, an essential aspect of learning is the ability to review written notes (see [21] discussed below). Captions could offer a dual benefit: they could improve access to a live lecture but also serve as a valuable tool for reviewing and rehearsing content and terminology when saved as written notes. Finally, specialistic terminology, whether in the STEM disciplines or the humanities, may lack direct sign language equivalents. In such cases, captions may provide clearer, faster, and more accessible alternatives to fingerspelling, which poses challenges even for highly proficient signers.

A clear example is the specialized language of mathematics, which presents a unique challenge due to its inherent complexity. For DHH students, this complexity often hinders the accurate extraction of key information from word problems [22]. Providing literal translation of mathematical problems, rather than a conceptual interpretation with an appropriate grammatical structure, may further complicate the translation process. In general, the language of mathematics is more challenging to interpret in sign language [23]. Interpreters often need to appeal to dactylology (or manual alphabet) to “spell” in sign language the nomenclature of concepts and/or procedures, or agree beforehand with the students on signs that can be used as a representation of these concepts and/or procedures. In either case, in the domain of mathematics, students with hearing impairment using sign language effectively experience a trilingual environment: the written language, the sign language, and the language of mathematics [24]. This further reinforces the potential advantage of captions, particularly in those academic settings where precise terminology is critical.

### 4.2. Related Studies Excluded from the Review

Two studies excluded from the review brief merit consideration [21,25]. Stinson et al. [21] was excluded because it did not comprise a “captions + sign language” condition in the experimental design; Debevc, Milošević, and Kozuh [25] was excluded because it did not involve an educational context, and yet it contrasted specifically captions plus sign language with sign language alone.

Stinson et al. [21] addressed the role of C-Print vs. sign language interpreting as a means to support accessibility to spoken lectures delivered through videos. In addition, they examined the additional role of study notes in content learning. The key finding from Stinson et al. [21] was that high school students retained more lecture information when they viewed speech-to-text support, compared to interpreter support. Instead, for university students, no difference between the two visual accessibility solutions emerged. The benefits of speech-to-text could be diminished by the greater educational maturity and experiential knowledge that university students tend to possess, particularly in relation to their familiarity with a diverse array of information formats. Two additional findings were noteworthy: first, retention was related to reading proficiency; second, DHH secondary school students in the delayed review group retained more information compared to students in the no-review and in the immediate review groups. In the context of the considerations above (Section 4.1), this study is relevant for three reasons. First, it shares a similar methodology with the included studies (i.e., severe-to-profound deafness, users with preference for sign language, assessment of reading skills, presentation of the educational content in video format, assessed content learning; see Table 2 and Table 3). Second, it reinforces the possibility that captions may be valuable for DHH students, even those who are proficient signers, as detailed in Section 4.1. Finally, it indicates that captions not only aid comprehension during a lecture but also provide valuable written notes for future review and the rehearsal of lecture content and terminology. Enhanced reading competencies empower DHH students to use educational materials more effectively after class, regardless of whether those resources are generated by captioning or provided by a note-taker. However, it should also be noted that the context of this research was confined to a conventional liberal arts lecture environment, which may not accurately represent alternative educational situations, such as experimental environments or instructional sessions that encompass complex material with symbolic representations. Again, these results would need appropriate empirical follow-up to be further confirmed and expanded.

Debevc, Milošević, and Kozuh [25] contrasted captions plus sign language with sign language alone, but outside an educational context and considering a broader range of hearing loss difficulties. The participants’ task was to observe four different educational videos on a diversity of themes (weather forecast, economy, hiking, shopping, culture, sports). Two videos showed a sign language interpreter explaining the contents, and two videos presented the same linguistic content plus captioning. Participants replied to multiple-choice questions about the contents of the videos. When captions were added to sign language interpreting, comprehension scores were higher. However, this effect emerged for hiking and culture themes, whereas no statistically significant difference emerged for shopping and sports themes. This study is relevant for two reasons. First, it adds to the limited body of empirical research on the combined effect of simultaneous captions and sign language. Second, the documented variations in outcomes across themes suggest that captions may not be consistently effective across all topics. As Debevc et al. [25] suggested, this could be due to differences in reading vocabulary. This finding calls for caution when assessing the relevance of captions and provides the methodological indication to probe different content domains when planning experiments on this issue.

### 4.3. Directions for Future Research

The limited number of studies identified in our review precludes strong practical implications for educational settings. Likewise, they are too few to suggest a meaningful conceptual framework for integrating multiple visual accessibility modalities. In what follows, we attempt to indicate possible domains where future research should focus to better understand these practical implications, especially in the context of ongoing technological advancements. It also suggests areas that need expansion to outline a better conceptual framework for discussing the coexistence of captions and sign language. Below, we propose three directions of research that merit attention.

#### 4.3.1. Attentional Conflict and Cognitive Load in the Real Classroom Scenario

As anticipated in Section 1, a primary concern regarding the possible coexistence of captions and sign language interpreting is the risk of attentional competition when processing multiple visual information sources, and the resulting cognitive load from accessing multiple streams of information. When conceptualized within the *attention networks framework* proposed by Posner and Petersen [26], the task of extracting content information from captions and sign language involves multiple dimensions of attention. First, because relevant information can be extracted from multiple sources, the DHH student must continuously monitor these visual streams and select which one to attend to at any given instance. This would engage the *orienting network*, responsible for the movement of attention throughout space. Depending on the relative position and distance of captions and sign language interpreting, this could entail small shifts in attentional resources in space (with or without eye movement involved, i.e., overt or covert), or large re-orienting behavior involving eye, head, or even trunk movements. Second, the need to prolong this visual task for tens of minutes, if not hours, as typically is the case in educational settings, would require the motivation and capability to sustain attention over the course of time. This would engage the *alerting network*, responsible for maintaining a physiological state of elevated sensitivity to sensory inputs. Finally, to the extent that two sources of information provide contradictory contents (e.g., mismatch in the semantic or syntactic content conveyed through sign language and captions), the *executive attention network* would be called into play, to detect and resolve any conflict between the incoming input and the expectations of the observer.

While anticipating the involvement of these multiple attentional networks is relatively straightforward, it is much more complex to understand if and how specific DHH learners might differ in their ability to engage these networks when processing captions and sign language. Although visual attention in deafness is a well-studied topic (for early reviews, see [27,28]; for a recent systematic review developed within the attentional networks framework, see [29]), almost all existing studies share three methodological limitations with respect to the issues discussed in the present article. First, they were conducted in laboratory settings (not classroom scenarios); second, they involved participants processing simple visual stimuli (not visual language); and third, they featured limited or no meaningful interaction between the visual sources (unlike the use of two concurrent streams conveying related linguistic and educational content). For these reasons, extrapolating clear predictions from existing studies regarding how the different visual abilities of deaf students might operate in real learning scenarios could prove largely speculative. This is especially true when considering that these attention skills specific to deafness change from childhood to adolescence (e.g., [30,31]) and may reflect the interactions between auditory deprivation and language deprivation that can sometimes occur in DHH students [32]. Nonetheless, at least two findings seem more robust than others and should be considered when discussing the possible coexistence of captions and sign language in educational settings. First, the difficulties that can emerge in vigilance and sustained attention (also referred to as “tonic alertness”; e.g., [33,34,35,36]); second, the documented advantage in responding to rapid events capturing attention, especially in the periphery of the visual field (e.g., [36,37]).

Beside these considerations related to attentional processing in students with deafness, it also remains to be ascertained if and how pairing captions with sign language interpreting could change the visual listening effort for the student. In spoken language processing, listening effort is understood as the listener’s engagement level with the task. According to the Framework for Understanding Effortful Listening (FUEL; [38]), when faced with a difficult acoustic environment (e.g., background noise), the subjective effort a listener reports is a function of two factors: the motivation to allocate resources and the consequent cognitive load required to successfully complete the listening task. On the one hand, there is evidence that visual listening effort can increase in deaf people processing sign language under more demanding visual conditions, such as comprehending signers who wear face masks [39,40]. On the other hand, it is also possible that the benefit of extracting complementary linguistic information from multiple sources (rather than subtracting facial information as in the context of face masks) could help students to sustain their motivation to allocate resources to the task of understanding and learning, possibly counteracting any cognitive load that may stem from the requests on attention we have outlined above.

In sum, while it is easy to see the potential challenge for attention and the potential burden in terms of cognitive load and visual listening effort for a DHH student that needs to monitor multiple sources of visual information while learning, it remains an empirical question to what extent these challenges come into play in real-class scenarios. Although some authors have attempted to extrapolate implications for learning environments (e.g., [41,42]), it is difficult to draw firm conclusions on how attention will operate in DHH students during classroom activities based on laboratory studies alone. Better understanding of how the learner selects information across the different information channels (e.g., captions, sign language interpreter, but also lip movements and slides) would greatly benefit from online eye and head movement measurements, which could directly inform about the explicit orienting of attentional resources in the visual scene (e.g., [43]). Moreover, it would be of prime importance that these experiments occur in real class scenarios [44] or, at the very least, in simulated real class scenarios [45], rather than just video presentations.

#### 4.3.2. A Broader Perspective on the Students with Deafness

The studies discussed in this article focus mostly on high school or university students, thereby constraining the generalizability of the findings to a wider demographic of DHH students, especially those with diverse educational experiences or requirements. It cannot be assumed that all DHH students possess the necessary language skills to fully benefit from these accessibility solutions. More proficient DHH readers comprehend captions better than those with lower reading proficiency, and captioning speed also influences comprehension [46,47,48]. Similarly, while some DHH individuals have strong sign language skills, linguistic proficiency within the DHH population varies substantially [49]. The high fluency needed by signers to have the best possible access to learning should not be underestimated. As studies have pointed out, the comprehension of signed lectures by deaf participants is normally below that of hearing participants receiving the lectures auditorily [50]. If signed comprehension is limited, then the placement of an interpreter in educational settings may turn out to be an insufficient measure for deaf people to reach educational levels similar to those of their hearing peers.

It is also fundamental to always keep in mind that the classroom is a complex social and cognitive environment, and sign language interpreters should be capable of blending linguistic fluency with pedagogical and developmental knowledge [51,52]. When interpreter training occurs outside of rigorous academic and expert-led institutions, the results can be inadequate. For instance, research in the US indicates that approximately 60% of the interpreters evaluated using an instrument used to assess and certify classroom interpreting (the Educational Interpreters Performance Assessment or EIPA [53]) had inadequate skills to provide full access for DHH signers [52]. To safeguard the educational rights of the DHH community using sign language, interpreter training must be coordinated within each nation, within specialized academic frameworks capable of ensuring the highest quality of professional competence.

With respect to the studies reported in Table 2 and Table 3, it should also be noted that the studies reported in Table 2 and Table 3 only included students with severe-to-profound deafness, having very limited or no access to auditory information. This scenario is now changing in many industrialized countries, as the availability of hearing assistive devices (such as cochlear implants or digital hearing aids) is transforming the classroom experience from a visual-only one into an audio–visual one. Future studies should thus also examine the potential of concurrent captions and sign language for students who have audio–visual access to classroom information. Extending these considerations and experiments to include deaf and hard-of-hearing (DHH) learners who use assistive devices will surely call for a reexamination of conclusions related to the deployment of attentional resources, as visual attention is likely to operate differently in the presence of auditory cues (e.g., [54,55]).

Finally, all included studies exclusively involved participants whose deafness or being hard-of-hearing was not associated with other sensory or cognitive deficits (e.g., low vision or neurodevelopmental difficulties). Since these associations do occur in DHH students [56], the relative contribution of captioning and sign language must also be examined in the context of these additional challenges.

#### 4.3.3. Captions and Sign Language in the Context of Emerging Technologies

The use of captions and sign language—either in combination or separately—in current educational settings has also been challenging due to practical obstacles, including severe budgetary restrictions, shortages of qualified support staff (such as interpreters), and the requirement for comprehensive professional development to ensure the adequate adoption and utilization of new technologies by existing personnel. However, technological advancements may change this scenario in the next decade. In expanding research on the possible advantages or disadvantages of using captions and sign language in combination, research should also consider these emerging technologies and their potential to overcome the current resource and cost limitations. Below, we discuss these accessibility improvements. However, it is important to bear in mind that even documented accessibility improvements do not necessarily imply a direct, positive effect on learning outcomes.

A first major technological advancement concerns the automatic conversion of speech into text or sign language. Speech-to-text transcriptions are already a reality, as we have all witnessed with the rise in online communication following the COVID-19 pandemic. Although automated transcripts still contain more errors compared to transcripts produced by a human professional [57], rapid progress in Large Language Models (LLMs) is expected to refine speech-to-text systems by improving semantics and syntactic predictions, potentially reducing transcription errors even further. Unlike speech-to-text transcriptions, speech-to-sign language translation is not yet available [58,59]. The main reason resides precisely in the fact that this conversion entails a translation (e.g., from Italian sign language to Italian written language), rather than transcription (e.g., from spoken to written Italian). Yet, the field of sign language recognition has evolved considerably over the last decade from early methodologies which primarily relied on Convolutional Neural Networks (CNNs) to classify static hand shapes [60] to recent vision transformers which can capture the global context of signs [61]. Moreover, attempts have been made to use wearable sensors to capture muscle signals and facial expressions in addition to visual information alone [62]. While these technological advancements are promising, the development of successful artificial systems for sign language generation (as well as sign language recognition and translation) requires an interdisciplinary effort involving experts in linguistics, natural language processing, computer vision, and computer graphics, along with members of the signing community [63]. High-quality datasets for sign language are far less available than those for spoken language, further complicating the training and development of robust AI models. Finally, a key challenge in sign language generation is the animation of virtual signers (avatars). The current technology still struggles to produce natural-looking signs and achieves a sub-optimal rendering of critical non-manual components of sign languages, such as facial expressions and body movements [59].

A second line of technological advancement worth considering is the increasing accessibility of low-cost augmented reality (AR) devices, which hold the potential to better integrate captions and sign language interpretation with the user’s line of sight, likely enhancing accessibility and engagement. The term “augmented reality” refers to the enrichment of real-world environments with digital contents superimposed onto the scene. In the context of this discussion, AR offers the potential to allow students to actively participate in real-world settings (e.g., classrooms) while receiving additional visual information through dedicated wearable devices such as head-mounted displays or smart glasses. This approach could ensure that assistive visual inputs remain within the user’s field of view, even as their head and eye position change. Several studies and technical reports have recently explored this opportunity (for examples see: [64,65,66,67,68]). The typical implementation involves light-weighted smart glasses that allow users to fully engage with the environment while simultaneously projecting in the visual field of the person captions, a sign language interpreter, or a combination of the two. Preliminary research has examined the feasibility (e.g., [67]) and the acceptability of these solutions ([66]).

A third avenue of technological support involves better optimization of the pacing of visual information provided to DHH students. As previously noted, the cognitive load that could result from concurrent visual information can be a challenge to learning, because it may limit the students’ ability to engage with the lecture and integrate the novel contents with prior knowledge. One solution to this cognitive overload is better segmentation of the information into manageable chunks (see also [2,3]). For instance, research indicates that students understand multimedia explanations better when they can control the pacing of the information, compared to when they experience the same contents as a continuous flow [69]. Mayer and Moreno [70] refer to this phenomenon as the segmentation effect. In practice, technology could facilitate the coordination of visual elements—such as captions, images, or diagrams—with the linguistic input from the sign language interpreter. This coordination can be achieved through pre-determined pauses between information segments, or through pacing that is contingent upon feedback from the learner or the interpreter. An example application of this idea is the SlidePacer system [71]. SlidePacer aims to facilitates real-time synchronization between the instructor and the interpreter via a Bluetooth-mediated protocol. Unlike a traditional presentation software, SlidePacer uses a managed transition state: upon a “next slide” command, the system holds the current slide in view until the interpreter signals via a mobile app that the translation is complete. This is followed by a configurable “visual processing delay”, ensuring that DHH students have sufficient time to process the slide content before the lecture proceeds.

In sum, while automatic captioning could become an accessibility tool for DHH individuals within the coming years, fully functional speech-to-sign language automatic interpreting remains a relatively more distant goal, likely out of reach for at least another decade. In the future, AR technology could also allow on-demand access to captions and sign language interpretation in contexts where such accessibility solutions are not routinely provided, or where users may prefer more discrete access to avoid stigma. Moreover, it offers a cognitive–perceptual advantage: the opportunity to address the issue of information dispersion across multiple locations in the visual scene. Finally, the possibility of better coordination between the instructor, the sign language interpreter, and the learner through interconnected technology could help better the segmentation of the visual contents. The key question for future empirical research related to educational contexts, however, is to what extent accessibility improvements related to emerging technology will result in demonstrated effects on learning outcomes for DHH students.

## 5. Conclusions

This review examined whether the concurrent use of captions and sign language improves or hinders content learning for DHH students in educational settings, compared to using these solutions separately. The small number of studies that emerged from this review precludes any firm conclusions for educational settings, and several aspects of the reviewed studies (differences in age, task features, and individual characteristics) limit their generalization.

As a final note, it is important to emphasize that the lack of evidence we describe in this article pertains only to the advantage/disadvantage of simultaneously using captions and sign language in educational settings, as revealed though empirical works. By no means does it challenge the established fact that sign language and spoken language can coexist and be utilized in conjunction with one another without compromising the integrity or efficacy of either mode of communication [9,10]. In fact, this combined experience of sign language and spoken language can significantly mitigate the potential risks associated with language deprivation in DHH children [32].

## Figures and Tables

**Table 1:** Search Strings.

Database	Combination of Title, Abstract, Keyworks and/or MeSH Terms Used
PubMed	((“Sign Language”[MeSH Terms] OR “captions”[Title/Abstract]) AND (“Education of Persons with Hearing Disabilities”[MeSH Terms]));
EMBASE	(‘deaf education’/exp OR ‘deaf education’:ti OR ‘deaf education’:ab AND ‘sign language’/exp OR ‘sign language’:ti OR ‘sign language’:ab OR ‘signed language’:ti OR ‘signed language’:ab OR ‘signing’:ti OR ‘signing’:ab OR ‘captioning’:ti OR ‘captioning’:ab OR ‘subtitles’:ti OR ‘subtitles’:ab OR ‘caption*’:ti OR ‘caption*’:ab OR ‘subtitl*’:ti OR ‘subtitl*’:ab);
PsychInfo	(DE captioning or captions or subtitles OR XB captioning or captions or subtitles OR DE sign language OR XB sign language OR DE sign language interpreters OR XB sign language interpreters) AND (DE deaf education OR XB deaf education OR DE deaf education and children OR XB deaf education and children).

**Table 2:** Characteristics of the sample and the context in each of the included studies.

Study	N	Degree Hearing Loss	SL Use	Reading Proficiency	Mean Age (Range) in Years	Context
Gentry et al. [17]	25 (DHH)	Not specified (participants described as “deaf students”)	SL users (primary communication mode by self report)	Adequate for the taks (as assessed by the tanford Achievement Test for the Hearing-Impaired, SAT-HI)	12.3 (8–18)	Elementary to Secondary-School
Marschark et al. [18], Exp.1	95 (DHH), 32 (NH)	80 dB or worse in the better ear (for 90% of students)	SL users (recruited from bilingual institute)	Adequate for the task (as assessed by ACT reading comprehension subtest)	N/A (estimate: 18–20 based on education level)	University
Marschark et al. [18], Exp.2	60 (DHH), 20 (NH)	80 dB or greater (for 93% of participants)	SL users (recruited from bilingual institute)	Adequate for the task (as assessed by ACT reading comprehension subtest)	N/A (estimate: 18–20 based on education level)	University
Marschark et al. [18], Exp.3	15 (DHH)	108 dB or greater in the better ear	SL users (recruited from bilingual institute)	Adequate for the task (as assessed by reading comprehension test)	N/A (12–16)	Secondary school
Marschark et al. [18], Exp.4	48 (DHH)	90 dB or greater in the better ear	SL users (recruited from bilingual institute)	Adequate for the task (as assessed by the teachers)	14.9 (N/A)	Secondary school
Yoon & Kim [19]	62 (DHH)	90 dB or greater in the better ear	SL users (first language by self-report)	Adequate for the task (as assessed by Test of Proficiency in Korean, TOPIK)	23.0 (N/A)	Training institute
Almalhy [20]	54 (DHH)	71 dB or greater in the better ear, all with unaided hearing	SL users (as assessed by the author)	Adequate for the task (as assessed by the author)	Not specified (estimate: 17–19 based on education level)	University

**Table 3:** Characteristics of experimental design and results.

Study	Stimuli	Conditions (Independent Variables)	Measures (Dependent Variables)	Outcome	Effect Size
Gentry et al. [17]	Video story	Print only vs. print + pictures, vs. print + SL, vs. print + pictures + SL (within-participant)	Accuracy in story retelling (in sign language)	**Print + pictures or Print + Pictures + SL** > Print only or Print + SL	Large (~48%)
Marschark et al. [18], Exp.1	Video lecture	C-Print, vs. SL, vs. C + SL (between-participant)	Accuracy in written multiple-choice questions	**C-Print** > SL or C-Print + SL	Moderate to large (~12%)
Marschark et al. [18], Exp.2	Video lecture (with slide presentation)	C-Print, vs. CART, vs. SL (between-participant)	Accuracy in written multiple-choice questions	No significant difference	N/A
Marschark et al. [18], Exp.3	Video lecture	Captions, vs. SL, vs. caption + SL (within-participant)	Accuracy in content questions	No significant difference	N/A
Marschark et al. [18], Exp.4	Video lecture	Caption vs. caption + SL (between-participants)	Accuracy in written multiple-choice questions	**Captions + SL** > Captions	Small, not confirmed by follow-up analyses
Yoon & Kim [19]	Video lecture	SL vs. Caption + SL (between-participants)	Accuracy in written multiple-choice questions	**Caption + SL** > SL	Medium to large (~55%)
Almalhy [20]	Video lecture	SL only vs. Captions only vs. SL + Captions (between-participants)	Accuracy in written multiple-choice questions on declarative and procedura knowledge	Declarative knowledge: **Captions** > SL > Captions + SL (immediate test); **Captions or SL** > Captions + SL (delayed test). Procedural knowledge: **SL** > Captions > Caption + SL (immediate test); **SL** > Captions > Captions + SL (delayed test).	Declarative knowledge: moderate to large (~78%, immediate test); (~27%, delayed test). Declarative knowledge: large (~54%, immediate test); (~52%, delayed test).

## Data Availability

Not applicable as no new empirical data was collected.

## Abbreviations

The following abbreviations are used in this manuscript:

DHH	Deaf or Hard-of-Hearing
UDL	Universal Design for Learning
CART	Communication Access Realtime Translation
D	Deaf
HoH	Hard-of-Hearing
C	Captioning
SL	Sign Language
LLMs	Large Language Models
AR	Augmented Reality
